# Evaluation of commercially available DNA extraction kits for the analysis of the broiler chicken cecal microbiota

**DOI:** 10.1093/femsle/fnz033

**Published:** 2019-03-27

**Authors:** Helga Pankoke, Irena Maus, Gunnar Loh, Andrea Hüser, Jana Seifert, Alexandra Tilker, Sarah Hark, Alexander Sczyrba, Stefan Pelzer, Jessica Kleinbölting

**Affiliations:** Evonik Nutrition & Care GmbH, Kantstraße 2, 33790 Halle, Germany; Computational Metagenomics, Faculty of Technology, Bielefeld University, Universitätsstrasse 25, 33615 Bielefeld, Germany; Evonik Nutrition & Care GmbH, Kantstraße 2, 33790 Halle, Germany; Evonik Nutrition & Care GmbH, Kantstraße 2, 33790 Halle, Germany; Institute of Animal Science, University of Hohenheim, Emil-Wolff-Str. 6–10, 70599 Stuttgart, Germany; Evonik Nutrition & Care GmbH, Kantstraße 2, 33790 Halle, Germany; Evonik Nutrition & Care GmbH, Kantstraße 2, 33790 Halle, Germany; Computational Metagenomics, Faculty of Technology, Bielefeld University, Universitätsstrasse 25, 33615 Bielefeld, Germany; Evonik Nutrition & Care GmbH, Kantstraße 2, 33790 Halle, Germany; Evonik Nutrition & Care GmbH, Kantstraße 2, 33790 Halle, Germany

**Keywords:** 16S rRNA gene amplicon sequencing, microbiome, chicken, cecum, DNA extraction kit

## Abstract

16S rRNA gene amplicon sequencing is a state of the art technology to analyze bacterial communities *via* microbiome profiling. Choosing an appropriate DNA extraction protocol is crucial for characterizing the microbial community and can be challenging, especially when preliminary knowledge about the sample matrix is scarce. The aim of the present study was to evaluate seven commercial DNA extraction kits suitable for 16S rRNA gene amplicon sequencing of the bacterial community of the chicken cecum, taking into account different criteria such as high technical reproducibility, high bacterial diversity and easy handling. The DNA extraction kits differed strongly with respect to extractable DNA quantity, DNA quality, technical reproducibility and bacterial diversity determined after 16S rRNA gene amplicon sequencing and subsequent bioinformatic and biostatistical data processing. While some of the DNA extraction protocols under-represented specific bacterial community members, the removal of PCR inhibitors supported technical reproducibility and subsequently enhanced the recovered bacterial diversity from the chicken cecum community. In conclusion, the removal of PCR inhibitors from the sample matrix seemed to be one of the main drivers for a consistent representation of the bacterial community even of low abundant taxa in chicken cecum samples.

## Teaser

An accurate description of the broiler caecal microbiota is a challenging tasks starting from a suitable DNA extraction to get insights into this complex habitat that affects animal health.

## INTRODUCTION

Our understanding of complex microbial ecosystem has significantly improved with the advent of 16S rRNA gene based sequencing approaches. These technologies take advantage of the presence of conserved and variable regions in the 16S rRNA gene, allowing sequence-based taxonomic analyses of bacterial ecosystems (Zuñiga, Zaramela and Zengler 2017). Since the 16S rRNA amplicon sequencing approach is well suited to give insights into the taxonomic composition of complex microbiomes, it has often been the method of choice to study the correlation of process parameters and community composition. However, this approach has known shortcomings. One of the main issues of taxonomic and functional profiling studies is the choice of an appropriate DNA extraction protocol (Kennedy *et al*. 2016; Costea *et al*. 2017; Soliman *et al*. 2017). The chosen method thereby needs to ensure a high technical reproducibility, insofar as series of consecutively performed experiments actually determine the same core microbiome for a given sample matrix.

The evaluation of DNA extraction kits and phylogenetic diversity on the microbial community composition has been investigated for many years (Starke *et al*. 2014; Albertsen *et al*. 2015; Fouhy *et al*. 2016; Gerasimidis *et al*. 2016). Previous studies that used a microbiome profiling approach have not only shown that extraction of DNA may substantially influence the outcome of the respective microbial community characterization (Wesolowska-Andersen *et al*. 2014; Tanase *et al*. 2015; Costea *et al*. 2017; Zielińska *et al*. 2017). They also indicated that the choice of the 16S rRNA gene variable region for the differentiation of microbial taxa (Burbach *et al*. 2015; Fouhy *et al*. 2016; Rintala *et al*. 2017), the applied sequencing technology (Fouhy *et al*. 2016; Allali *et al*. 2017) and finally the bioinformatics pipelines (Allali *et al*. 2017) shape the taxonomic community profiles. Studies of the gastrointestinal tract (GIT) of chicken or pigs additionally showed that its microbial composition is mainly affected by the efficiency of cell lysis instead of DNA recovery (Anderson and Lebepe-Mazu 2003; Scupham, Jones and Wesley 2007). Other studies reported that the overall effect of DNA extraction on the 16S rRNA gene based gut microbiota profile was relatively small, whereas the 16S rRNA gene target region had an immense impact on the results (Rintala *et al*. 2015).

The GIT of chicken has received significant attention due to results of issues that relate to food safety, feed conversion rate, animal nutrition and health (Yeoman *et al*. 2012; Borda-Molina, Seifert and Camarinha-Silva 2018). The GIT of broiler chicken harbors a complex microbiome. Besides archaea, fungi and viruses, the microbial community mainly consists of bacteria (Yeoman *et al*. 2012) that significantly affect bird health and performance in chicken meat production (Rinttilä and Apajalahti 2013). The ceca comprise the most diverse and abundant bacterial community of all segments of the chicken GIT (Gong *et al*. 2007), representing an important GIT section where the microbial fermentation is most active (Yeoman *et al*. 2012). Due to the high technical variability of microbiome profiling approaches, it is still difficult to compare microbiome data from different studies and to exactly define microbiome signatures that may be indicative for the health status or performance level of broilers.

In the present study, we investigated whether different DNA extraction protocols affected the composition of broiler cecal microbiota that was determined with the established method of high-throughput 16S rRNA gene amplicon sequencing on the Illumina MiSeq system. DNA was extracted from pooled cecal contents of ten healthy broiler chickens using seven commercial DNA extraction kits with the aim to determine a commercial kit suitable to reproducibly characterize the cecal bacterial community. The kits differed with respect to using thermal and/or mechanical lysis, the duration of the respective extraction steps as well as an optional final filtering step to remove PCR inhibitors. To assess the impact of DNA extraction protocol on the representation of the cecal microbiome, we compared DNA quantity and quality, and focused on technical reproducibility and bacterial diversity, which is a good proxy for overall protocol performance and accuracy of recovered abundance profiles (Costea *et al*. 2017). Finally, all DNA extraction protocols were evaluated with respect to their respective strengths and weaknesses for bacterial community profiling of the broiler chicken cecum microbiome. Our working hypothesis was that DNA extraction protocols that either used mechanical and/or thermal lysis differed in their ability to extract DNA quantity (Burbach *et al*. 2015; Barbosa *et al*. 2016; Costea *et al*. 2017). Furthermore, as chicken feed is essentially composed of plant material that contains factors that might inhibit the amplification of nucleic acids by PCR (Wilson 1997), we expected that a cleaning step such as the removal of PCR inhibitors from the sample matrix might improve DNA quality, technical reproducibility and subsequently bacterial taxonomic profiles.

## MATERIAL AND METHODS

### Animal trial, chicken intestinal sample collection and extraction of total microbial DNA

The animal experiment was carried out in the Agricultural Experiment Station of Hohenheim University, located in Lindenhöfe in Eningen (Germany), in strict accordance with the German Animal Welfare legislation. All procedures regarding animal handling and treatments were approved by the Animal Welfare Commissioner of the University (HOH40/16TE). Ten Ross 308 broilers from the experimental trial described in Siegert, Helmbrecht and Rodehutscord (2017) were randomly chosen. After 22 days, the birds were anesthetized by a gas mixture and euthanized by carbon dioxide (CO_2_) exposure. After opening the ceca and removing the digesta material with a sterile spatula, digesta samples of the ten birds were pooled, homogenized and stored at −80°C until further processing.

After homogenization of the cecum material, four aliquots per DNA extraction procedure (28 samples in total) were transferred to Eppendorf reaction tubes (5 mL). Total DNA was extracted from the cecum material using seven commercial DNA extraction kits according to manufacturer's instructions (Table 1). For each kit, we additionally included one blank sample to be able to remove potential background contamination from the used kits (Salter *et al*. 2014). All kits are recommended for DNA extraction from different matrices such as from bacteria, yeast, algae, fungi, soil or feces. We determined DNA quantity of the extracted DNA using the Invitrogen Qubit 4 Fluorometer (Thermo Fisher Scientific, Waltham, MA USA) using Qubit dsDNA BR Assay Kit 100 pg. DNA quality was determined *via* the A260/280 and A260/A230 ratios using Nanodrop (Thermo Fisher Scientific, Waltham, MA USA). Thereby, the A260/A280 ratio indicates protein contamination, while the A260/A230 ratio refers to non-nucleic acid contaminants such as residues of salts or solvents.

**Table 1.** Overview over the seven commercial kits used for DNA extraction.

No	DNA extraction kit	Manufacturer	Thermal lysis	Mechanical lysis	PCR inhibitor removal	Proteinase K treatment	Biomass used for DNA extraction [mg]	Elution volume [µL]
1	innuPREP Stool DNA kit	Analytic Jena, Jena, Germany	15 min/95°C, 900 rpm and additional incubation with mutanolysin for 2.5 h	—	—	20 min/70°C	250	50
2	ISOLATE Fecal DNA kit	Bioline, London, United Kingdom		2 × 40 s Precellys Bashing Beat Lyse Tube	—	—	150	40
3	FastDNA™ Spin kit for soil	MP Biomedicals, Santa Ana, CA, USA		2 × 40 s Fast Prep Homogenizer Bead-MatrixE	—	—	250	30
4	PSP^®^ Spin Stool DNA kit	Stratec, Berlin, Germany	10 + 3 min at room temperature/95°C, 11,000 rpm	Vortex for 2 min, 5 Zirconia Beads II	InviAdsorb Tube	10 min/70°C, 900 rpm	200	100
5	NucleoSpin^®^ DNA Stool kit	Machery & Nagel, Düren, Germany	5 min/70°C	Vortex for 10 min, NucleoSpin Bead Tubes Type A	NucleoSpin Inhibitor Removal	—	220	100
6	QIAamp^®^ DNA Stool Mini kit	Quiagen, Hilden, Germany	5 min/70°C	—	InhibitEX tablet	10 min/70°C, 900 rpm	220	55
7	PowerSoil^®^ DNA Isolation kit	MoBio, Carlsbad, CA, USA	—	Vortex for 10 min, Power Bead Tubes	Solution C2 is patented Inhibitor Removal Technology (IRT)	—	180	55

### Microbial community structure analysis by high-throughput 16S rRNA gene amplicon sequencing

The microbial community of pooled cecum samples was taxonomically characterized by 16S rRNA gene amplicon sequencing applying the 16S metagenomic sequencing library preparation protocol (Illumina Inc., 2014) for sequencing library construction. To amplify the third and fourth variable regions (V3, V4) of the 16S rRNA gene, we used bacterial primers 341F and 785R as described by Klindworth *et al*. (2013). The obtained amplicon libraries were sequenced on the Illumina MiSeq system (Illumina, CA, USA) with a total sequencing output of about 10 million read pairs using the paired-end protocol (300 bp paired-end read, V3 chemistry) at LGC Genomics (Berlin, Germany). For amplicon processing, libraries for each sequencing lane were demultiplexed by Illumina bcl2fastq 1.8.4 software. Raw reads were sorted by amplicon inline barcodes and adapters were clipped. Reads with a final length < 100 bases were discarded. In addition, primer sequences were detected and clipped allowing three mismatches per primer. Subsequently, high-quality sequences were merged using BBMerge 34.48 (http://bbmap.sourceforge.net/). Finally, data were validated using FastQC (Andrews 2015).

Data pre-processing and OTU picking with Mothur version 1.35.1 (Schloss *et al*. 2009) was performed as follows: a) removal of sequences containing ambiguous bases (Ns), with homopolymer stretches of more than 8 bases or with an average Phred quality score below 3, b) alignment against 16S Mothur-Silva SEED r119 reference data, c) filtering of short alignments (truncated or unspecific PCR products), d) sequence subsampling to 60,000 sequences per sample, e) sequencing error reduction by pre-clustering (up to 1 differing base per 100 bases allowed in a cluster), f) elimination of chimera with the uchime algorithm (Edgar *et al*. 2011), g) taxonomical classification of the sequences against the Silva reference classification (database release r119 as of 24 July 2014) and removal of sequences from other domains of life, h) OTU picking by clustering at the 97% identity level and i) OTU consensus taxonomical calling, integrating the taxonomical classification of the cluster member sequences. Finally, these processing steps resulted in the creation of an OTU count table showing taxonomic distributions.

### Data filtering and biostatistical analyses

After bioinformatic processing of the sequence data, we applied consecutive filtering steps to the OTU table. First of all, potential bacterial background contamination specific for each commercial kit was removed by using the respective blank sample (Salter *et al*. 2014). Furthermore, OTUs with at least 10 read counts per OTU cluster, which also occurred in at least two samples, were retained in the data set. Finally, OTUs that could not be classified at phylum level were removed from the dataset. Data filtering reduced the number of OTUs to 1006, of which the average number of read counts per sample was 37 872 (min: 26 948, max: 45 976). Amplicon data were then rarefied to 26 933 sequences using *rrarefy()* from the R package *vegan* (Oksanen *et al*. 2015), which reduced the number of OTUs from 1006 to 1003.

To analyze the effect of DNA extraction protocol (treatment) on DNA quality and quantity, we performed non-parametric Kruskal–Wallis tests. As post-hoc test, we used pairwise comparisons with the Mann–Whitney U-test based on a normal approximation for *P*-value calculation. To account for false positives, the obtained *P*-values were corrected for the false discovery rate (Benjamini and Hochberg 1995). To estimate the influence of DNA extraction protocol on the bacterial community composition, multivariate statistics were performed on the rarefied OTU table. We performed a hierarchical cluster analysis (‘average linkage’ and ‘Bray–Curtis distance’) and non-metric multidimensional scaling (NMDS) using the function *metaMDS*() with default settings from the package *vegan*. We additionally compared the OTU composition of the two clusters derived from the cluster analysis with an ANOSIM.

To estimate bacterial alpha diversity, diversity indices such as Species Richness, Pielou's Index of Evenness and the Shannon diversity Index were calculated using the corresponding functions implemented in *vegan*. We analyzed the effects of DNA extraction protocol on bacterial diversity with Kruskal–Wallis tests and post-hoc tests as described above. To further determine whether DNA extraction protocols from cluster 1 differed from cluster 2 DNA extraction protocols (see Results section) with respect of diversity scores, Mann–Whitney U-tests were performed as described above.

For visual inspection of taxonomic composition of the bacterial community with stack bars, the read counts of all four technical replicates per DNA extraction protocol were summed up. For each of the seven DNA extraction protocols, the read counts of all OTUs belonging to one specific taxon were again summed up and normalized by the total number of read counts. Each taxonomic level was then deduced from the normalized OTU counts by summing up the classifications on different levels. For visualization of the taxonomic groups, the respective proportions of the different taxa were ranked by decreasing value. Taxa with less than 0.5% of prevalence were summed up and were being classified as of ‘low prevalence’.

To furthermore determine taxa that differed significantly between the two main clusters (see Results section), we performed a permutational multivariate analysis of variance (perMANOVA) using the Bray–Curtis distance matrix implemented in the package *vegan* and pairwise comparisons with T-tests for all taxonomic levels from ‘phylum’ to ‘genus’. Again, *P*-values were corrected for multiple testing as described above.

For each parametric analysis, normality and variance homogeneity were analyzed with the Shapiro-Wilk test and the Bartlett test, respectively. All statistical analyses were performed using R version 3.3.3 (R Developmental Core Team 2017). In the boxplots, the median is the bold horizontal line, the boxes refer to the interquartile range, and whiskers extend to max. 1.5 times the interquartile range, whereas circles are outliers.

## RESULTS

### Determination of amount and purity of extracted DNA

**Figure 1.**
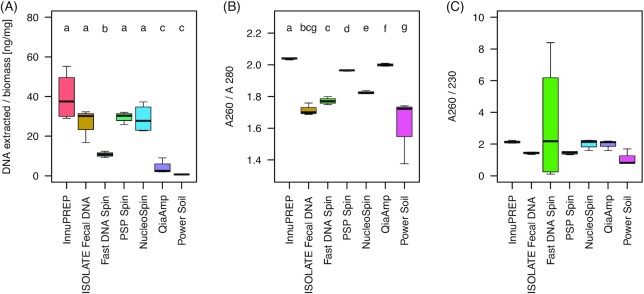
DNA quantity (determined with Qubit) and DNA quality (determined with Nanodrop) of DNA from chicken caecum content that was extracted with seven commercial DNA extraction kits. **A)** Total DNA extracted per used amount of biomass, **B)** A260/280 (pure DNA ∼ 1.8) and **C)** A260/230 (uncontaminated DNA ∼ 2.0). Different letters above the boxplots indicate significant differences between the used DNA extraction protocols (pairwise Mann–Whitney U-tests with P fdr ≤ 0.05) after significant Kruska–Wallis tests (*P* ≤ 0.05); n = 4 replicates per treatment). Definition of boxplots is given in Materials and Methods. Abbreviations: innuPREP—innuPREP Stool DNA kit; ISOLATE Fecal DNA—ISOLATE Fecal DNA kit; Fast DNA Spin—FastDNATM Spin kit for soil; PSP Spin—PSP® Spin Stool DNA kit; NucleoSpin—NucleoSpin® DNA Stool kit; QIAamp—QIAamp® DNA Stool Mini kit, PowerSoil—PowerSoil® DNA Isolation kit.

The DNA extraction methods differed significantly with respect to the absolute amounts of DNA extracted per mg biomass (Fig. 1A; Kruskal–Wallis test, df = 6, chi² = 22.49, *P* < 0.001), and DNA quality (Fig. 1B-C; A260/A280: chi² = 25.87, *P* < 0.001; A260/A230: chi² = 12.33, *P* = 0.055). Two of the seven kits, namely the PowerSoil® DNA isolation kit and the QIAamp® DNA Stool Mini kit, provided very low amounts of DNA (< 5 ng/mg biomass). With respect to DNA quality, only the NucleoSpin^®^ DNA Stool kit had a A260/A280 ratio close to ∼1.8, which is generally accepted as ‘pure’ and suitable for further analysis. Three other kits, namely the innuPREP Stool kit, PSP^®^ Spin Stool DNA kit and QIAamp^®^ DNA Stool Mini kit, had values close to ∼2.0, indicating a slight contamination (Fig. 1B). The samples extracted with the PowerSoil^®^ DNA Isolation kit protocol showed the lowest A260/A280 including some technical variation (Fig. 1B). The A260/A230 ratio of the FastDNA™ Spin kit for soil showed huge inconsistencies within the four replicates, indicating contamination of salts or solvent residues in some of the technical replicates (Fig. 1C).

### Biostatistical characterization of the bacterial cecum community

**Figure 2.**
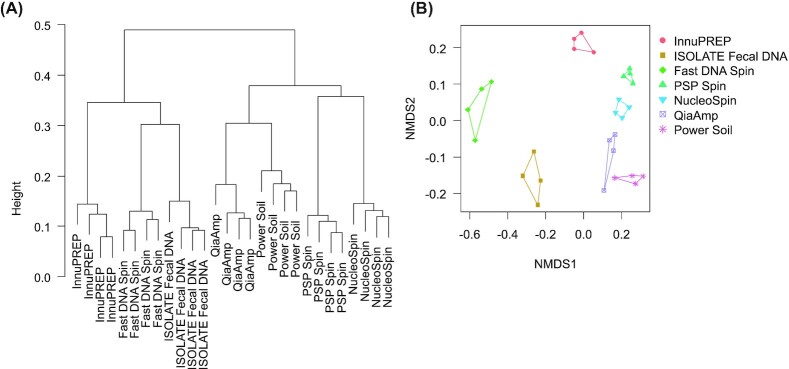
**A)** Hierarchical cluster analysis (‘Bray–Curtis distance‘ and ‘average linkage‘) and **B)** NMDS (NMDS with ‘Bray–Curtis distance’). Multivariate analyses were based on 1003 OTUs after rarefaction (read counts per sample: 26,933; n = 4 replicates per treatment). Abbreviations: innuPREP—innuPREP Stool DNA kit; ISOLATE Fecal DNA—ISOLATE Fecal DNA kit; Fast DNA Spin—FastDNATM Spin kit for soil; PSP Spin—PSP® Spin Stool DNA kit; NucleoSpin—NucleoSpin® DNA Stool kit; QIAamp—QIAamp® DNA Stool Mini kit, PowerSoil—PowerSoil® DNA Isolation kit.

To investigate the effect of DNA extraction protocol on the representation of the cecal bacterial community, we performed multivariate statistical analyses. A hierarchical cluster analysis showed that the DNA extraction protocols clustered in two groups (Fig. 2A). Group 1 comprised the innuPREP Stool DNA kit, the ISOLATE Fecal DNA kit and the FastDNA™ Spin kit for soil, whereas a second cluster contained the QIAamp^®^ DNA Stool Mini kit, PowerSoil^®^ DNA Isolation kit, PSP^®^ Spin Stool DNA kit and NucleoSpin^®^ DNA Stool kit. The four technical replicates of each DNA extraction protocol clustered together. NMDS ordination confirmed the high similarity within the four technical replicates per treatment and furthermore showed that the main variation between the DNA extraction protocols was related to the presence or absence of a PCR inhibitor removal step along the first axis (Fig. 2B; ANOSIM for cluster 1 versus cluster 2: R = 0.917, *P* = 0.001).

**Figure 3.**
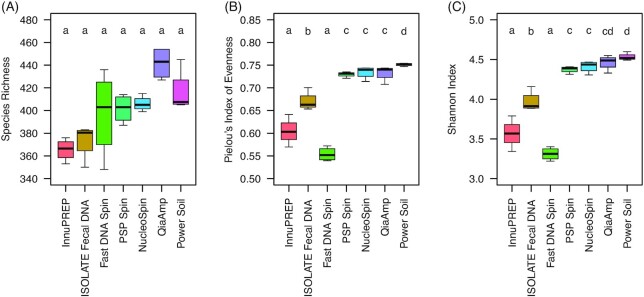
Bacterial diversity in chicken caecum content extracted with seven commercial DNA extraction kits. **A)** Species Richness, **B)** Pielou's Index of Eveness and **C)** Shannon Index. Alpha diversity indices were estimated based on 1003 OTUs using rarefied data (read counts per sample: 26,933). Different letters above the boxplots indicate significant differences between the DNA extraction protocols (pairwise Mann–Whitney U-tests with *P* fdr ≤ 0.05 after significant Kruskal-Wallis tests (*P* < 0.01); n = 4 replicates per treatment). Definition of boxplots is given in the Materials and Methods section. Abbreviations: innuPREP—innuPREP Stool DNA kit; ISOLATE Fecal DNA—ISOLATE Fecal DNA kit; Fast DNA Spin—FastDNATM Spin kit for soil; PSP Spin—PSP® Spin Stool DNA kit; NucleoSpin—NucleoSpin® DNA Stool kit; QIAamp—QIAamp® DNA Stool Mini kit, PowerSoil—PowerSoil® DNA Isolation kit.

DNA extraction protocols also strongly affected bacterial diversity (Fig. 3A-C), which was particularly evident for the Pielou's Index of Evenness and the Shannon Index (Fig. 3B–C, Kruskal–Wallis test, df = 6, chi² = 24.84, *P* < 0.001 and chi² = 24.53, *P* < 0.001), but not so much for Species Richness due to higher variation between replicates (Fig. 3A, chi² = 18.25, *P* < 0.01). Furthermore, DNA extraction protocols belonging to cluster 1 had significantly lower diversity scores than those from cluster 2. For example, the median number of OTUs found by cluster 1 protocols was on average 8% lower than the number of OTUs found by cluster 2 protocols (average ± SD CL1: 379 ± 26, CL2: 416 ± 20, Mann–Whitney U-test, W = 23.5, *P* < 0.001). Moreover, samples extracted with kits from cluster 2 had a higher evenness (average ± SD CL1: 0.61 ± 0.05, CL2: 0.74 ± 0.01, Mann–Whitney U-test, W = 0, *P* < 0.001) and a higher Shannon diversity (average ± SD CL1: 3.6 ± 0.03, CL2: 4.4 ± 0.09, Mann–Whitney U-test, W = 0, *P* < 0.001).

### Taxonomic profiling of a bacterial cecum community

**Figure 4.**
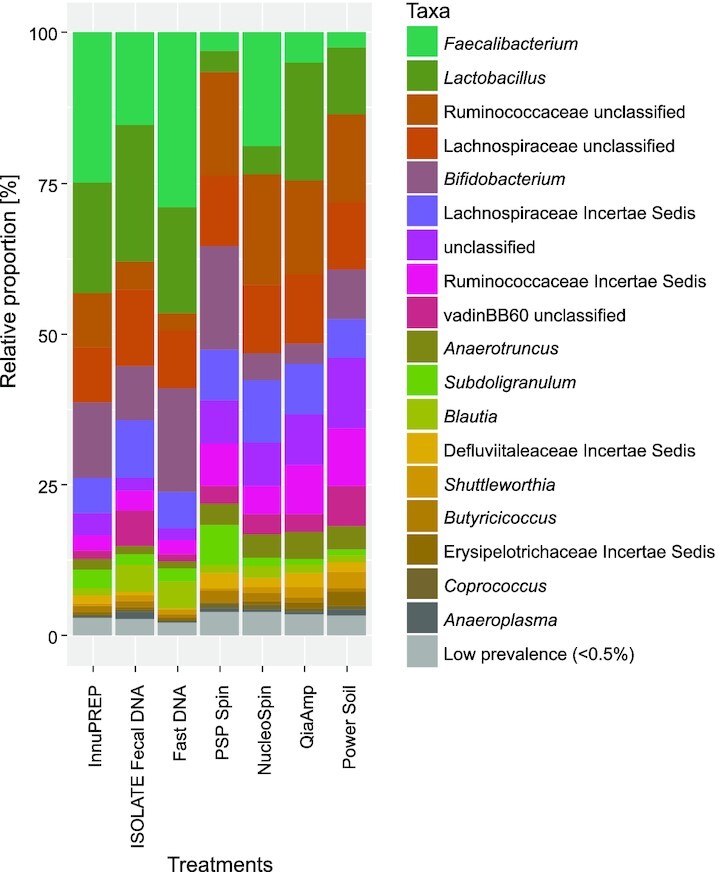
The stack bar shows the most abundant genera of the bacterial community of the chicken caecum based on 1003 OTUs using rarefied data (read counts per sample: 26,933). All taxa were consistently found by all seven commercial DNA extraction kits used. Taxa displayed correspond to the relative distribution of OTUs (for which the read counts of the 4 replicates per treatment were summed up). Low prevalence corresponds to <0.5% of the respective taxon relative to the absolute number of read counts per treatment. The order of the taxa in the legend reflects the relative average abundance of the respective taxa over all seven treatment groups. Abbreviations: innuPREP—innuPREP Stool DNA kit; ISOLATE Fecal DNA—ISOLATE Fecal DNA kit; Fast DNA Spin—FastDNATM Spin kit for soil; PSP Spin—PSP® Spin Stool DNA kit; NucleoSpin—NucleoSpin® DNA Stool kit; QIAamp—QIAamp® DNA Stool Mini kit, PowerSoil—PowerSoil® DNA Isolation kit.

Taxonomic classification of the cecum bacterial community showed that the phylum *Firmicutes* dominated the microbial composition of all analyzed samples, followed by *Actinobacteria* and *Tenericutes* (Fig. S1, Supporting Information). On class level, the majority of the bacteria in the samples belonged to *Clostridia* (Min-max: 64%–88%), *Bacilli* (4%–23%)—both taxa are *Firmicutes*—and *Actinobacteria* (3%–17%), phylum *Actinobacteria*). The most abundant orders were *Clostridiales* (63%–88%)*, Lactobacillales* (4%–23%) and *Bifidobacteriales* (3%–17%). The most abundant families identified within the phylum *Firmicutes* were *Ruminococcaceae* (28%–51%)*, Lachnospiraceae* (17%–29%), and *Lactobacillaceae* (4%–23%), while among *Actinobacteria*, the family *Bifidobacteriaceae* (4%–17%) occurred most frequently (Fig. S2, Supporting Information). Each of the seven DNA extraction protocols detected the 18 main genera that occurred with more than 0.5% prevalence (Fig. 4). Thereby, *Faecalibacterium* (3%–29%), *Lactobacillus* (4%–23%), unclassified *Ruminococcaceae* (3%–18%), unclassified *Lachnospiraceae* (9%–13%)*, Bifidobacterium* (3%–17%) and *Lachnospiraceae incertae sedis* (6%–10%) dominated the microbial community (Fig. 4). These taxa comprised approximately 80% of the cluster 1 microbiomes, whereas they made up for only 60% of cluster 2 microbiomes. Of the 30 genera present with less than 0.5% prevalence, cluster 2 kits found on average 28 whereas cluster 1 kits found only 25 of the low abundant genera.

Further comparative analyses of the different taxonomic levels based on relative read counts revealed that the microbiome represented by cluster 1 and cluster 2 kits differed on family and genus level (perMANOVA for phylum, class, order: *P* > 0.05; perMANOVA for ‘family’: F_1,5_ = 2.8, R² = 0.36, *P* < 0.05; perMANOVA for ‘genus’, F1,5 = 7.7, R² = 0.61, *P* < 0.05). The post-hoc analysis for the level ‘family’ showed that kits from cluster 1 and 2 did not differ from each other (T-test, *P* fdr > 0.05). On ‘genus’ level, kits from cluster 2 had significantly higher read counts of *Ruminococcaceae* unclassified at genus level (T-test, *P* fdr < 0.05), of taxa unclassified below order level (‘unclassified’; *P* fdr < 0.05) and of the genus *Anaerotruncus* (*P* fdr < 0.01) (Fig. 4). The comparison of all seven DNA extraction kits with each other revealed multiple differences in extraction efficiency for particular taxa, e.g. the families *Ruminococcaceae* (Kruskal–Wallis test, *P* fdr < 0.05), *Lachnospiraceae* (*P* fdr < 0.05) and *Lactobacillaceae* (*P* fdr < 0.05) (Fig. S2, Supporting Information).

## DISCUSSION

Characterization of a microbial community using cultivation-independent approaches depends on accurately assessing the corresponding microbiome (see e.g. Costea *et al*. 2017). Yet even though microbiome analyses nowadays are state of the art, it still remains a challenge to correctly describe the entire microbiome of a given sample matrix. In this study, we evaluated the performance of seven commercial DNA extraction kits on broiler cecal microbiota applying high-throughput 16S rRNA gene amplicon sequencing for community characterization. Even though we used one pooled sample, from which all technical replicates were generated, DNA quantity and DNA quality differed severely between the seven DNA extraction kits used (Fig. 1A–C). Previous studies on microbiome characterization have shown that mechanical lysis (Burbach *et al*. 2015) or the combination of mechanical and thermal lysis (Barbosa *et al*. 2016) provided enhanced DNA quantity. In the present study however, high DNA yield did not automatically correlate with the form of lysis applied (mechanical or thermal or both) (Fig. 1A). The DNA yield also did not seem to affect diversity estimates later on, as kits from cluster 1 that in general yielded higher amounts of DNA (> 10 ng/mg biomass) nevertheless had lower diversity scores than the respective kits from cluster 2 (Fig. 3A–C). Also DNA quality did not seem to affect bacterial diversity or the 16S rRNA gene derived OTUs as much (Fig. 1B–C, Fig. 3A–C). However, a high variation of the A260/A230 ratio indicating contamination with salts or solvent of some replicates of the FastDNA™ Spin kit might have increased the variation of Species Richness (Fig. 1C and Fig. 3A). This result is not unexpected, as salts or solvent residues in the DNA are known to interfere with PCR.

Multivariate analyses furthermore showed that the OTU composition of samples derived from DNA extraction protocols with a PCR inhibitor removal step were highly similar to each other, as were those missing this step (Fig. 2A,B), indicating a low within-treatment variation and a higher between-treatment variation. In general, diversity indices determined in the present study were rather high, even for previously tested kits, when compared to other GIT systems (Siegert, Helmbrecht and Rodehutscord 2017). In accordance with our expectations, all commercial kits that used a cleaning step to remove PCR inhibitors from the extracted DNA (cluster 2 kits) had higher bacterial diversity scores than the three commercial kits without additional cleaning step (cluster 1 kits) (Fig. 3A–C). As bacterial diversity is a good proxy for overall protocol performance and accuracy of recovered abundance profiles (Costea *et al*. 2017), our results suggest that the removal of potentially PCR-interfering substances might substantially increase the quality of metagenomic profiling studies in general. As chicken feed mainly consists of plant material such as soy, corn or wheat that might contain huge quantities of secondary plant compounds known to interfere with PCR (Wilson 1997), the removal of the respective compounds from the DNA extracts made from chicken gut content might even be essential for all PCR-dependent sequencing technologies.

In general, the taxonomic composition of the chicken cecum content determined in the present study by 16S rRNA gene based taxonomic profiling resembled previous studies (e.g. reviewed in Borda-Molina, Seifert and Camarinha-Silva 2018). Of the prevalent genera, *Faecalibacterium* might be related to bird age at sampling (Oakley *et al*. 2014). Most importantly, all taxa with more than 0.5% prevalence were consistently found by all used DNA extraction kits (Fig. 4). However, the seven DNA extraction protocols and in particular cluster 1 vs. cluster 2 kits differed with respect to the proportional representation of the main bacterial taxa of the cecal microbiome (Fig. 4). Even though at higher taxonomic levels differences were not that apparent, proportional differences were most pronounced at genus level, where DNA extraction protocols from cluster 1 underrepresented some bacterial taxa. Furthermore, the removal of potential PCR inhibitors increased the probability to find low abundant taxa. Previous studies have reported differences with respect to the taxonomic representation of a sample matrix by different DNA extraction protocols (Burbach *et al*. 2016; Costea *et al*. 2017). Most probably, the observed differences in the present study occurred due to the removal of PCR inhibitors included in cluster 2 protocols, which might have allowed the PCR primers to binding the corresponding DNA fragment more efficiently. However, we are aware of the fact that this bias could occur to many other aspects in addition to DNA extraction efficiency. For example, biases during PCR amplification or the *rrn* gene copy number could also affect the PCR (Farrelly *et al*. 2015). Thus, to properly test our hypothesis, future studies should both isolate DNA according to the manufacturer's instructions and also by omitting the step of removing the inhibitors using the same protocols.

Yet even though DNA extraction protocols that removed PCR inhibitors seemed to be more similar to each other than the protocols without this cleaning step (Fig. 2A and B and Fig. 4), the four cluster 2 kits still revealed significant differences with respect to the proportional microbial community composition. For example, the PSP® Spin Stool and NucleoSpin® Stool kits under-represented the *Lactobacillaceae* in comparison to the other two cluster 2 kits and the three cluster 1 kits (Fig. S2, Supporting Information). The genus *Lactobacillus* mostly consists of rod-shaped, Gram-positive and highly lysis-resistant bacteria e.g. *Lactobacillus casei* (Nagaoka *et al*. 1990; Alimolaei and Golchin 2016). In general, mechanical lysis and bead beating increase the extraction efficiency of Gram positive bacteria (Costea *et al*. 2017). Our data do not support this general notion, as only those two kits that both used thermal and mechanical lysis underrepresented the Lactobacillaceae. However, both kits better extracted Ruminococcaceae, which are also Gram-positive bacteria, indicating that the combination of thermal and mechanical lysis might competitively favor the extraction of particular Gram-positive bacterial taxa over others.

In conclusion, our results suggest that a cleaning step, which removes PCR inhibitors, improves consistency and reproducibility for the 16S rRNA gene amplicon technique in particular and thereby might provide a robust representation of the microbial diversity in this difficult type of matrix. Based on our results obtained for the chicken cecal community, we recommend cluster 2 kits for bacterial community characterization due to the combination of high DNA yield and efficient handling time, high technical reproducibility within replicates, high alpha diversity scores and increased sensitivity for low abundant taxa.

### Nucleotide sequence accession number

The 16S rRNA gene amplicon sequence data was deposited in the European Nucleotide Archive (ENA) under the study accession number PRJEB25932.

## Supplementary Material

fnz033_Supplemental_File
