# Supplementary material for: Evaluation of commercially available DNA extraction kits for the analysis of the broiler chicken cecal microbiota
Source: FEMS Microbiol Lett. 2019 Mar 27;368(8):fnz033. doi: 10.1093/femsle/fnz033 (PMC8112482; doi:10.1093/femsle/fnz033)
Supplement: fnz033_Supplemental_File [file fnz033_supplemental_file.docx]

**Supporting Information**

**
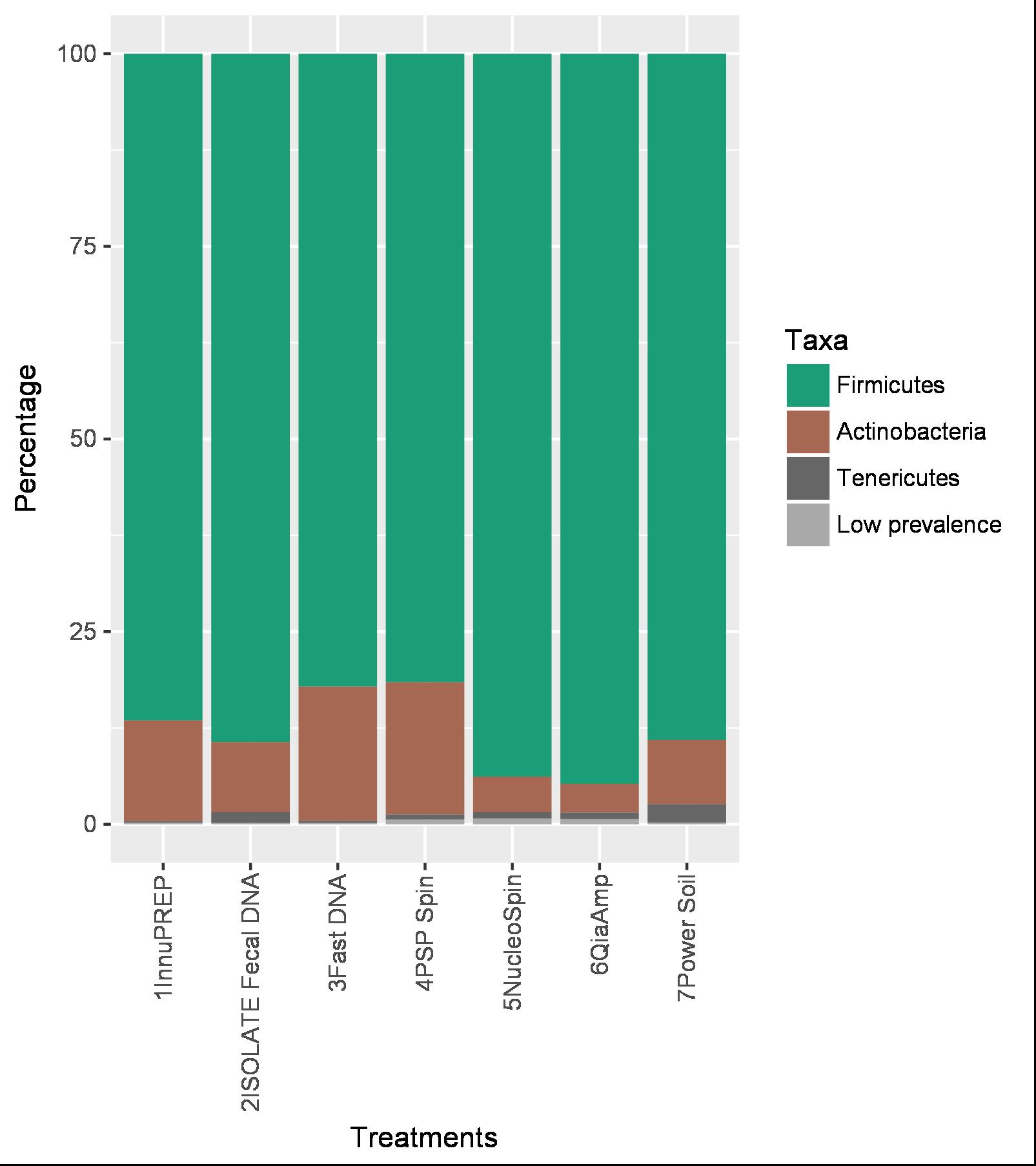
**

**Supplementary Figure S1**: The stack bar shows the most abundant phyla of the bacterial community of the chicken caecum, of which the DNA was extracted with seven commercial DNA extraction kits. Taxa displayed correspond to the relative distribution of OTUs (for which the read counts of the 4 replicates per treatment were summed up). Low prevalence corresponds to < 0.5% of the respective taxon relative to the absolute number of read counts per treatment. The order of the taxa in the legend reflects the relative average abundance of the respective taxa over all seven treatment groups. Abbreviations: innuPREP - innuPREP Stool DNA kit; ISOLATE Fecal DNA - ISOLATE Fecal DNA kit; Fast DNA Spin – FastDNA^TM^ Spin kit for soil; PSP Spin - PSP® Spin Stool DNA kit; NucleoSpin - NucleoSpin® DNA Stool kit; QIAamp - QIAamp® DNA Stool Mini kit, PowerSoil - PowerSoil® DNA Isolation kit.

**
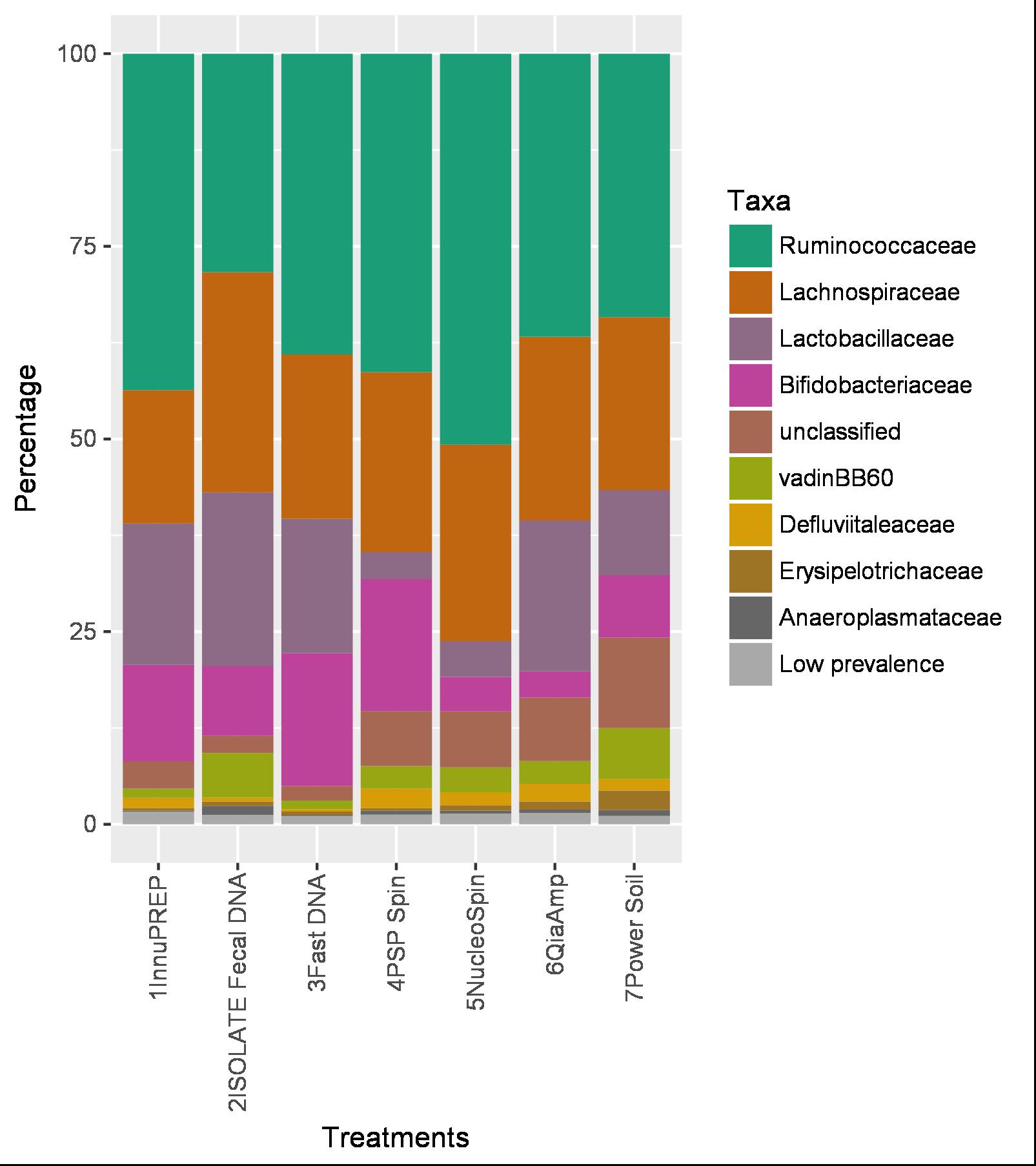
**

**Supplementary Figure 2:** The stack bar shows the most abundant families of the bacterial community of the chicken caecum, of which the DNA was extracted with seven commercial DNA extraction kits. Taxa displayed correspond to the relative distribution of OTUs (for which the read counts of the 4 replicates per treatment were summed up). Low prevalence corresponds to < 0.5% of the respective taxon relative to the absolute number of read counts per treatment. The order of the taxa in the legend reflects the relative average abundance of the respective taxa over all seven treatment groups. Abbreviations: innuPREP - innuPREP Stool DNA kit; ISOLATE Fecal DNA - ISOLATE Fecal DNA kit; Fast DNA Spin – FastDNA^TM^ Spin kit for soil; PSP Spin - PSP® Spin Stool DNA kit; NucleoSpin - NucleoSpin® DNA Stool kit; QIAamp - QIAamp® DNA Stool Mini kit, PowerSoil - PowerSoil® DNA Isolation kit.
